# Can We Reboot the Role of Intraperitoneal Chemotherapy in the Treatment for Gastric Cancer with Peritoneal Carcinomatosis?: A Retrospective Cohort Study Regarding Minimally Invasive Surgery Conjoined with Intraperitoneal plus Systemic Chemotherapy

**DOI:** 10.3390/cancers14092334

**Published:** 2022-05-09

**Authors:** Sungho Kim, Chang-Min Lee, Danbi Lee, Jong-Han Kim, Sungsoo Park, Seong-Heum Park

**Affiliations:** 1 Department of Surgery, Korea University College of Medicine, Seoul 02841, Korea; gskimsh0205@korea.ac.kr (S.K.); ppongttai@korea.ac.kr (J.-H.K.); kugspss@korea.ac.kr (S.P.); pshchw@korea.ac.kr (S.-H.P.); 2 Department of Surgery, Korea University Ansan Hospital, Ansan 15355, Korea; shedevil@korea.ac.kr; 3 Department of Surgery, Korea University Guro Hospital, Seoul 08308, Korea; 4 Department of Surgery, Korea University Anam Hospital, Seoul 02841, Korea

**Keywords:** gastric cancer, peritoneal carcinomatosis, minimally invasive surgery, intraperitoneal chemotherapy

## Abstract

**Simple Summary:**

The prognosis of gastric cancer (GC) patients with peritoneal carcinomatosis (PC) remains poor, in spite of the recent evolution of systemic chemotherapy for patients with GC. Nowadays, as intraperitoneal (IP) chemotherapy has been applied in the treatment for GC patients with PC, several promising data have been suggested. Inspired by the known characteristics of minimally invasive surgery (MIS), we have recently applied MIS concept in IP and systemic chemotherapy for GC patients with PC. We retrospectively compared the clinical outcomes between the patients underwent MIS conjoined IP plus systemic chemotherapy and patients underwent systemic chemotherapy only, to show the promising potential of the new treatment strategy combining MIS concept with IP plus systemic chemotherapy for GC patients with PC.

**Abstract:**

Background: Peritoneal carcinomatosis (PC) is the most common form of metastasis in gastric cancer (GC) and is related with a poor prognosis. Several treatment modalities including systemic chemotherapy and intraperitoneal chemotherapy have been studied and adopted in treatment of GC patients with PC. Nevertheless, few studies have reported the comparison of the oncologic outcomes between minimally invasive surgery (MIS) with intraperitoneal (IP) chemotherapy and conventional chemotherapy for GC with PC. Methods: We retrospectively reviewed the clinical records of 74 patients who had been diagnosed as GC with PC via either intra-abdominal exploration or abdominopelvic computed tomography between January 2011 and April 2021. After performing propensity score-matching for this retrospective data, we compared the outcomes of 26 patients who underwent MIS followed by IP combined systemic chemotherapy (MIS-IP group) and 26 patients who underwent systemic chemotherapy only (SC-only group). Results: The 2-year progression free survival rate of the MIS-IP group was significantly higher than the SC-only groups (36.4% and 10.5%, respectively; *p* = 0.010). In multivariate analysis to detect relevant factors on PFS, IP chemotherapy (HR 0.213; *p* < 0.001), Eastern Cooperative Oncology Group performance status (HR 3.689; *p* = 0.002), and the amount of ascites (*p* = 0.011) were significant prognostic factors. Conclusions: This study demonstrated the therapeutic potential of MIS conjoined IP plus systemic chemotherapy for GC patients with PC. MIS conjoined by IP plus systemic chemotherapy can be adopted as a treatment option to reboot the role of IP chemotherapy in GC patients with PC.

## 1. Introduction

Gastric cancer remains one of the most common cancers worldwide and patients with unresectable or metastatic disease have dismal prognosis [1]. Peritoneal carcinomatosis (PC) is the most common form of metastasis in gastric cancer (GC), detected in up to 30% of advanced GC patients [2,3].

Although various treatment options are being studied to improve the prognosis of patients with an unresectable disease, palliative systemic chemotherapy is primarily recommended by the Japanese guidelines [4]. However, the response of systemic chemotherapy in metastatic patients is limited, especially in patients with PC [5].

Nowadays, as a treatment modality for PC, intraperitoneal (IP) chemotherapy has risen based on pharmacokinetic evidence of peritoneum-plasma barrier [3,6]. The safety and efficacy of IP administration of paclitaxel in ovarian cancer patients with PC have been verified by several clinical trials [7,8,9]. The pharmacologic features of paclitaxel [10,11,12] encouraged its application in gastric cancer; however, the application of IP chemotherapy in GC patients has demonstrated inconsistent results [13,14,15]. Recently, a multicenter randomized controlled trial (RCT) was carried out to evaluate the safety and clinical efficacy of IP paclitaxel in combination with S-1 and paclitaxel compared to standard systemic chemotherapy. This phase III study marginally failed to demonstrate a significant superiority of IP chemotherapy regimen over the standard regimen but suggested the potential clinical efficacy of IP chemotherapy [14].

Nevertheless, many researchers are still trying to update the treatment potential of IP chemotherapy for PC. In Korea, several prospective studies including PIPS-GC trial (Identifier KCT0004670) and IPLUS trial (Identifier KCT0003187) are ongoing to investigate the feasibility of IP chemotherapy. Our center has also cumulated the expertise of IP chemotherapy for PC. However, we have focused on the role of surgical resection as well as IP chemotherapy; therefore, have tried to reboot the role of surgical resection in the treatment for stage IV disease. This treatment concept started from the Sugarbaker et al. [16] strategy, in which cytoreductive surgery (CRS) and hyperthermic intraperitoneal chemotherapy (HIPEC) are mutually complementary in the treatment for PC cases. In 2010, Li et al. [17] showed the significance of HIPEC in the outcomes of 128 PC cases, but they also emphasized the role of surgical resection. 

Furthermore, since 2014, we have been inspired by the advantages of minimally invasive surgery (MIS) for advanced gastric cancer (AGC), which includes not only the fast recovery due to the small wounds, but also the highly informative image for the surgical field. From the expertise regarding MIS for AGC, we contemplated that the magnified view of MIS enables the operators to overcome the complicated conditions of stage IV disease. In addition, via the MIS approach, the patients with high peritoneal cancer index (PCI) score could undergo neoadjuvant intraperitoneal and systemic chemotherapy (NIPS) with the minimized surgical trauma [18].

The purpose of this study is to share our experiences including MIS approach conjoined with IP combined systemic chemotherapy in GC patients with PC, and analyze the safety and clinical outcomes compared to those of patients who underwent systemic chemotherapy only for GC with PC. 

## 2. Materials and Methods

### 2.1. Study Design and Participants

This study is a retrospective cohort study based on data from a single institution: the electronic medical charts of patients who were diagnosed with PC arising from gastric adenocarcinoma between January 2011 and April 2021. All the patients had been confirmed to be gastric cancer via endoscopic examination and to have undergone the preoperative staging via endoscopic ultrasound and abdominopelvic computed tomography (APCT) [19]. The patients in whom visceral metastasis had been diagnosed by APCT were excluded from this study. 

According to whether IP chemotherapy was accepted, the patients were divided into the following two groups: patients who underwent MIS conjoined with IP plus systemic chemotherapy (named as MIS-IP group) and those who underwent systemic chemotherapy only (named as SC-only group). The clinical outcomes were compared between the MIS-IP and SC-only groups. 

We obtained written informed consent from all participants for all procedures associated with the study. The study was approved by the Institutional Review Board of Korea University Medical Center Ansan Hospital (registration number: 2021AS0124).

### 2.2. Eligibility of MIS-IP Group

#### 2.2.1. Inclusion criteria

(i) Histologic proven gastric adenocarcinoma via endoscopic examination
(ii) Age between 20 and 85 years
(iii) Histologic proven peritoneal carcinomatosis via diagnostic laparoscopy
(iv) Eastern Cooperative Oncology Group performance status 0 or 1

#### 2.2.2. Exclusion Criteria

(i) Patients who refuse IP chemotherapy
(ii) Patients with visceral metastasis diagnosed by the initial APCT
(iii) Patients with metastatic recurrence after the prior radical gastrectomy
(iv) Patients who are not tolerable to the liquid diet or enteral feeding
(v) Patients with other malignancy

### 2.3. Details of IP Chemotherapy

**Figure 1:**
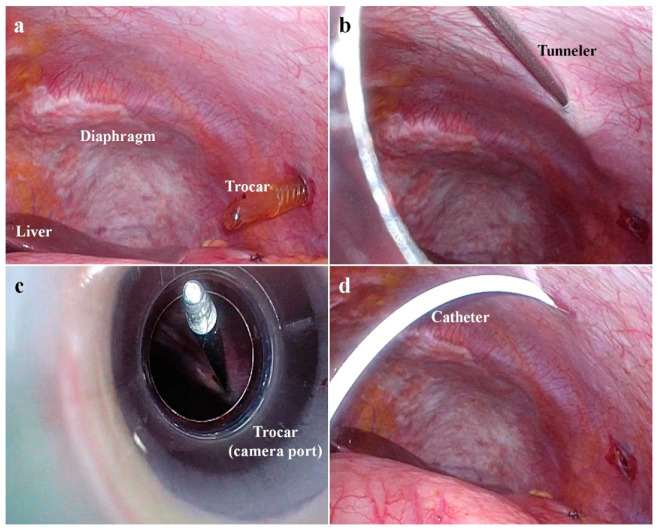
Insertion of a port system for intraperitoneal (IP) chemotherapy. (**a**) During the diagnostic laparoscopy, one of the working port sites (a trocar inserted) was selected as a port site for IP chemotherapy. (**b**) The trocar was removed from the working port site. Then, a tunneler was inserted through the port wound and penetrated the peritoneal wall beside the working port site. (**c**) The tunneler was extracted through the trocar of the camera port. (**d**) The tunneler-connected catheter was inserted into the abdominal cavity.

For the patients who underwent diagnostic laparoscopy, a peritoneal biopsy was performed to confirm PC in the intraoperative pathologic diagnosis. If PC was confirmed in the pathologic examination, a port system for IP chemotherapy (Healthport; Baxter Healthcare SA, Zurich, Switzerland) was implanted for every patient who agreed with undergoing IP chemotherapy. During the first diagnostic laparoscopy, regardless of whether surgical resection was performed or not, the port system for IP chemotherapy (Healthport; Baxter Healthcare SA, Zurich, Switzerland) was implanted in the subcutaneous fat layer at the right or left subcostal area (Figure 1).

IP chemotherapy using paclitaxel was started more than 1 week after diagnostic laparoscopy to prevent the fluid leakage from the wound, if diagnostic laparoscopy was not followed by non-curative surgical resection. Every second week (according to the schedule of FOLFOX chemotherapy) or every three weeks (according to the schedule of XELOX chemotherapy) week, 60 mg of paclitaxel diluted in 500 mL normal saline was administered over two hours and the remaining fluid was drained after 12 h.

### 2.4. Data Collection

We collected data on the age at the diagnosis, sex, body mass index (BMI), American society of anesthesiologists (ASA) score, ECOG-PS, serum CEA level, serum CA 19-9 level, the amount of ascites, the presence of the other organ metastasis, histology, Bormann classification, the information on non-curative surgery including the operation name and postoperative complications, information on chemotherapy including the first line chemotherapy agent, cycles and chemotherapy induced toxicity, progression, and mortality.

The amount of ascites was graded based on APCT scan: mild, ascites localized in only one area; moderate, ascites neither mild nor massive; and massive, ascites extended throughout the abdominal cavity [20]. Postoperative complications were graded according to the Clavien–Dindo classification (CDC) [21]. The initial bone marrow, liver, kidney function, and chemotherapy induced toxicity were determined and graded according to the common terminology criteria for adverse events (CTCAE) version 3.0 [22].

### 2.5. Statistical Analysis

The propensity score-matching (PSM) method was used based on binary logistic regression to minimize the selection bias caused by imbalanced variables in the historical cohort study. The variables were entered into the propensity score model including patients’ age, sex, ECOG-PS, serum CEA level, serum CA 19-9 level, the amount of ascites, and histology. One-to-one matching between the MIS-IP and SC-only groups was conducted using the nearest-neighbor matching method. The seed number was 74, and the value of caliper size was 0.2 in PSM.

Continuous variables are presented in mean ± standard deviation rather than median owing to the small sample size. Statistical differences were assessed using chi-square test or Fischer’s exact tests and Mann–Whitney U test for categorical and continuous variables, respectively. Kapalan–Meier method was used to compare the progression-free survival (PFS) between the MIS-IP and SC-only groups. The Cox proportional hazard model was used to investigate the significant factors affecting on PFS. Univariate analyses were performed for all variables in Table 1. Then, the significant variables in univariate analyses were included in multivariate analysis; variable selection was done using the backward elimination method with a likelihood ratio test. Statistical analysis was performed using SPSS version 25.0 (SPSS, Inc., Chicago, IL, USA) and any *p*-value < 0.05 was considered statistically significant. 

## 3. Results

After performing PSM to reduce the bias caused by the historical cohort study, the compositions of the demographics and baseline characteristics were statistically even between the MIS-IP and SC-only groups (Table 1). The mean age at diagnosis was 58.3 years. Based on ECOG, 61.5% was scored PS 0, and 38.5% was scored PS 1. The amount of ascites based on APCT was none to mild in 86.6% and moderate to massive in 13.4%. The details of the MIS-IP group are summarized in Table 2.

The clinical outcomes of the two groups are shown in Table 3. Twelve patients (46.2%) underwent non-curative gastrectomy (other than conversion surgery) and six (23.1%) underwent bypass surgery in the MIS-IP group, whereas two (7.7%) underwent non-curative gastrectomy and two (7.7%) underwent bypass surgery in the SC-only group. The incidence of surgery-related complications was not significantly different between the MIS-IP and SC-only group. Chemotherapy-induced toxicity did not differ between the two groups.

**Figure 2:**
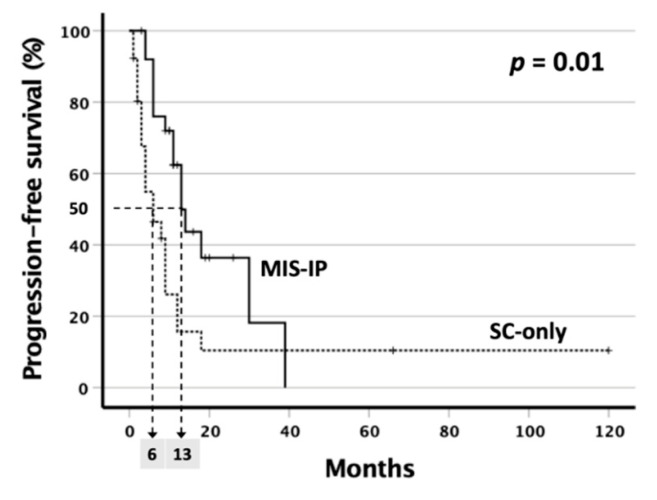
Kaplan–Meier survival curve for progression-free survival (PFS) in gastric cancer patients with peritoneal carcinomatosis.

Kaplan–Meier survival curve was constructed to compare the PFS between the two groups. The 2-year PFS rate was significantly higher in the MIS-IP group than the SC-only group (36.4% and 10.5%, respectively, *p* = 0.010)(Figure 2). The median PFS was also longer in the MIS-IP group than the SC-only group (13 and 6 months).

The 2-year PFS rate was significantly higher in the MIS-IP group than the SC-only group (36.4% and 10.5%, respectively, *p* = 0.010), and the median PFS was longer in the MIS-IP group than the SC-only group (13 and 6 months).

In the multivariate analysis, IP chemotherapy (HR 0.213, *p* < 0.001), ECOG PS (HR 3.689, *p* = 0.002), and the amount of ascites (*p* = 0.011) were revealed as the significant prognostic factors for PFS (Table 4).

## 4. Discussion

Some researchers have emphasized the value of surgical resection to reduce the tumor burden in stage IV gastric cancer [23,24,25,26]. However, in such advanced cases, non-curative gastrectomy embeds several issues. First, it is technically demanding to overcome the distorted anatomy of stage IV cancer, regardless of whether the surgical manner is curative or not. Surgeons should deal with the hard omental cake and ambiguous vascular structures covered with desmoplastic adhesions. These conditions are sometimes associated with unexpected accidents including massive bleeding or irreversible organ injury. Second, although we can achieve R2 resection in patients with PC, gastrointestinal reconstruction must be associated with the high risk of anastomotic or stump leakage. Regarding R1 resection, several studies reported that positive resection margin was not related with anastomosis leakage in gastric cancer surgery [27,28]. However, we dare not make any anastomosis involved with the seeding nodules in patients with stage IV disease. Finally, PC cases are sometimes associated with severe adhesions due to desmoplasia; therefore, we should take a risk of the unintended organ-injury during adhesiolysis. In particular, gastrointestinal reconstruction itself is not allowed due to the mesenteric contracture in the high PCI scored cases.

**Figure 3:**
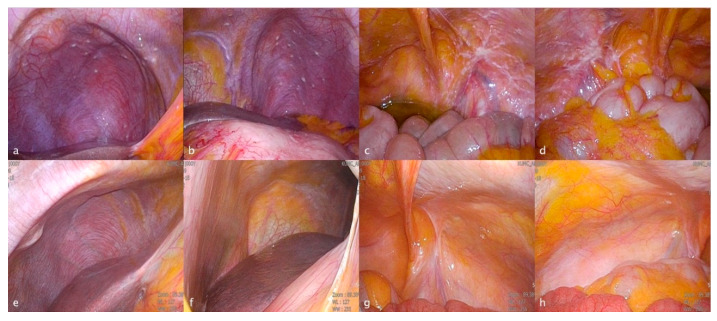
The laparoscopic images of a patient with peritoneal carcinomatosis before and after neoadjuvant intraperitoneal and systemic chemotherapy (NIPS). (**a**) Right upper quadrant view before NIPS. (**b**) Left upper quadrant view before NIPS. (**c**) Right lower quadrant view before NIPS. (**d**) Left lower quadrant view before NIPS. (**e**) Right upper quadrant view after NIPS. (**f**) Left upper quadrant view after NIPS. (**g**) Right lower quadrant view after NIPS. (**h**) Left lower quadrant view after NIPS.

Recent rise of IP chemotherapy is one clue for overcoming the hindrances mentioned above. As shown in this study, although we encountered PC from AGC, it was not more necessary to abandon “curative” treatment. IP chemotherapy, in which chemotherapeutic agents directly attack the tumor cells on the peritoneum, is expected to be effective for PC, whereas systemic chemotherapy is hindered by the blood peritoneal barrier [29]. Therefore, in cases where R2 resection is possible, IP chemotherapy could provide some possibility to resolve the residual lesions including PC. In addition, as shown in Figure 3, even when PC overwhelmed whole abdominal organs, NIPS enable us to acquire “a few” chances to try conversion surgery (case no. 24 in Table 2) [18].

Nevertheless, IP chemotherapy is not widely accepted yet, because little evidence exists in the current field of gastric cancer treatment [30,31,32]. In particular, PHOENIX-GC trial, which is the first RCT to compare combined IP and systemic chemotherapy with systemic chemotherapy in GC with PC, also failed to show the statistical superiority of IP plus systemic chemotherapy [14]. Thus, all patients with PC do not accept IP chemotherapy. In our institute, the patients accepting IP chemotherapy have been treated with combined IP and systemic chemotherapy for GC with PC, whereas ones who do not accept IP chemotherapy underwent systemic chemotherapy alone. These two treatment options were simultaneously suggested to the patients with PC; therefore, we could compare the clinical outcomes between the patients who underwent systemic chemotherapy alone and systemic plus IP chemotherapy.

Although our study showed the prognostic significance of IP chemotherapy, it is necessary to pay attention to the following issues as well as the promising aspects of IP chemotherapy itself. 

First, long-term survivors exist in both groups, either patients who underwent systemic chemotherapy only or MIS followed by IP plus systemic chemotherapy. Nowadays, even though many researchers have focused on the promising perspectives of IP chemotherapy, all PC cases do not show the dramatic response to IP plus systemic chemotherapy. In addition, it is not obvious to determine whether IP chemotherapy was more effective than systemic treatment in some patients who underwent combined IP plus systemic chemotherapy. Furthermore, in our institute, diagnostic laparoscopy showed PC regression in a few patients who underwent systemic chemotherapy only. Thus, it is rational that the other factors might be remarked as well as treatment modality in stage IV disease. In this study, other than IP chemotherapy, ECOG PS and the amount of ascites appeared as the independent prognostic factors for PFS; in general, a good ECOG PS has been considered to have a better prognostic impact than a poor one, [33,34,35] and the amount of ascites appeared as a sign for the poor nutritional condition and high possibility of metastasis in a recent study [36]. In other words, we cannot conclude that IP chemotherapy might cure all the patients with PC. This issue correlates with several limitations of the retrospective study regarding stage IV disease; the heterogeneity of chemotherapeutic agents used in each group, no stratification of PCI score in the SC-only group, and the small populations. In particular, PCI score is known as a significant disease-factor in the patients with PC; therefore, this limitation should be considered in interpreting our results. 

**Figure 4:**
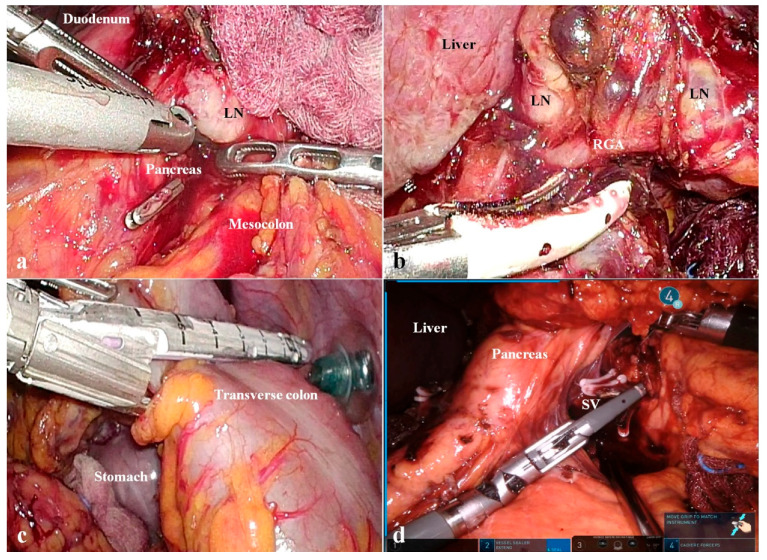
The images regarding non-curative gastrectomy for gastric cancer patients with peritoneal carcinomatosis. (**a**) A conglomerated lymph node was dissected from the pancreatic head and mesocolon using ultrasonic energy shears (LN, lymph node). (**b**) The huge lymph node was separated from the hepatic artery (LN, lymph node; RGA, right gastric artery). (**c**) The transverse colon abutted from the gastric tumor was resected using a laparoscopic linear stapler. (**d**) Under the pancreas, the splenic vein was divided using a bipolar vessel sealing device (SV, splenic vein).

Next, in this study, a laparoscopic or robotic approach was maintained for the patients who were undergoing non-curative gastrectomy or conversion surgery. Although the recent reports have also supported the oncologic safety of MIS for AGC, [37,38,39] the distorted anatomy of stage IV disease has been regarded as a high barrier to MIS. However, our center has cumulated the expertise regarding a minimally invasive approach for AGC [40,41]; therefore, we could perform a laparoscopic or robotic non-curative gastrectomy in stage IV disease. As shown in Figure 4, we have tried to get rid of whole tumor-involved tissues, as if the previous surgeons suggested CRS before. In other words, the purpose of our surgical procedure was to minimize all tumor burden other than PC. This strategy does not correlate with the classical treatment concept for stage IV disease; REGATTA trial, the first RCT to address the significance of reduction surgery in AGC, also denied the prognostic effect of palliative gastrectomy in stage IV disease [42]. However, CRS plus HIPEC, in which radical surgery reduces the tumor burden and IP chemotherapy eradicates PC, [16] does not correspond to the conventional treatment paradigm for stage IV disease. In a similar manner, we have performed a laparoscopic or robotic gastrectomy depending on the effect of IP chemotherapy as if CRS relies on HIPEC. Here, our discriminating strategy was to facilitate repeating IP chemotherapy using paclitaxel; this method correlated with the reason why we have adopted the MIS approach against the challenging conditions of stage IV disease. Due to the limited depth of peritoneal infiltration, paclitaxel is not appropriate for one-time bolus infusion of HIPEC [43]. However, paclitaxel is advantageous in long-term repeated administration, since it has the strong anti-proliferative activity that rarely makes intraperitoneal adhesions [14]. Regarding this issue, we expected that the MIS approach could contribute to the repeated IP paclitaxel chemotherapy, since laparoscopic or robotic procedures cause less adhesions than open surgery. In our center, a GC patient with PC could undergo 72 times of IP chemotherapy (case No. 9 of Table 2). Furthermore, the MIS approach using the newly developed gears was intended to update the surgical outcomes for the far advanced cases. Due to the technical advances, the surgical instruments have been continuously developed; therefore, the cutting edge devices enable the surgeons to overcome the dangerous anatomy of the highly advanced cases [40,41]. Additionally, the high-resolution image of the laparoscopic or robotic surgical system enhances the operator’s eyes in manipulating the distorted structures around the tumor. Therefore, MIS for stage IV disease might be correlated with the potential to extend the resectability as well as reducing the wound for the fast recovery. 

**Figure 5:**
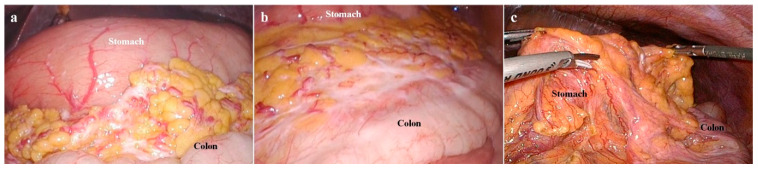
Conversion surgery after neoadjuvant intraperitoneal-systemic chemotherapy. (**a**) Omental cake was noted in the first diagnostic laparoscopy. (**b**) Omental cake was partially regressed in the second diagnostic laparoscopy (about six months after the first diagnostic laparoscopy). (**c**) Conversion surgery was performed in the final diagnostic laparoscopy, in which omental cake and peritoneal seeding was completely regressed (about 24 months after the first diagnostic laparoscopy).

Finally, the MIS approach was helpful in making a decision between non-curative resection and NIPS for the high PCI scored cases, in which a non-curative gastrectomy could not be performed. In the treatment of patients with PC, one important decision was whether a non-curative gastrectomy should be postponed or not. Regarding this issue, we contemplated that a laparoscopic approach was more appropriate than an open laparotomy. Most of all, the magnified view provided us with the detailed information regarding the tumor invasion; therefore, we could avoid the excessively invasive procedures (e.g., pancreaticoduodenectomy or vascular reconstruction) in stage IV GC. Although the advanced tumor can be more easily manipulated in open surgery than MIS, open procedure does not always guarantee the resectability in the highly advanced disease. Furthermore, since the ultimate purpose of NIPS was to reach the resectable disease status, in which surgical resection could be tried, [30,32,44] it might be advantageous that the MIS approach (e.g., laparoscopic biopsy or laparoscopic gastrojejunostomy) be performed as the precedent procedure of conversion surgery. As shown in Figure 5, laparoscopic procedures were possible at the time of conversion surgery, as the MIS approach might cause few adhesions throughout the three times of diagnostic laparoscopies (case no. 9 in Table 2). 

All these features make our treatment method (used in MIS-IP) different from CRS-HIPEC, which is another treatment strategy for GC patients with PC. Although both CRS-HIPEC and our method are indicated for the PC cases, it is impossible or difficult to perform CRS in some patients with a high PCI score: (1) severe PC involvement induces the mesenteric contraction; therefore, we cannot restore the continuity of the digestive tract in reconstruction process after palliative resection; (2) to complete CRS in cases of PC extensively involving the small bowels, we cannot avoid a dangerous anastomosis between the PC-involved organs; or (3) if we encounter the more severe PC cases, any manipulation cannot be performed due to the intra-abdominal adhesions. However, the treatment strategies used in the MIS-IP patients are flexible according to the PC status. If surgical resection is impossible or dangerous, we can choose NIPS in which IP and systemic chemotherapy are repeated till the time of conversion surgery. On the other hand, when palliative gastrectomy is possible (PC minimally involving the small bowels, the desmoplastic adhesion can be overcome by the meticulous dissection, or anastomosis expected to be free of tumor), surgical resection is adopted at the time of diagnostic laparoscopy. These strategies may contribute to avoiding the severe morbidity or mortality associated with the impellent surgery. Furthermore, as described above, MIS-IP strategies may increase the number of repeating IP chemotherapy by reducing the possibility of intra-abdominal adhesion; this characteristic is expected to be associated with the oncologic outcome, although we have yet cumulated the enough data to compare the survival data between CRS-HIPEC and MIS conjoined IP plus systemic chemotherapy.

## 5. Conclusions

In conclusion, this retrospective study demonstrated a promising potential of MIS conjoined IP plus systemic chemotherapy in the treatment for GC patients with PC. Although the retrospective data might embed some limitations in the comparative analysis regarding stage IV disease, MIS conjoined with IP plus systemic chemotherapy can be considered as a treatment option to reboot the role of IP chemotherapy in GC patients with PC. Therefore, this treatment modality is worth being re-evaluated in the prospective study, in which we can control the biases arising from the patient factors (i.e., ECOG PS or PCI score).

## Figures and Tables

**Table 1:** Comparison of demographics and baseline characteristics between the two groups.

Variables	Before Propensity Matching Analysis			After Propensity Matching Analysis		
	MIS-IP (*n* = 38)	SC-Only (*n* = 36)	*p*	MIS-IP (*n* = 26)	SC-Only (*n* = 26)	*p*
Age at diagnosis	56.9 ± 14.1	62.8 ± 13.9	0.071	57.9 ± 14.1	58.7 ± 12.2	0.843
Sex (%)			0.125			0.532
Male	21 (55.3)	27 (75)		18 (69.2)	20 (76.9)	
Female	17 (44.7)	9 (25)		8 (30.8)	6 (23.1)	
BMI	22.6 ± 2.9	21.3 ± 3.3	0.043	22.8 ± 3.1	21.6 ± 3.0	0.088
ASA classification (%)			0.532			0.699
I	10 (26.3%)	11 (30.6%)		6 (23.1%)	8 (30.8%)	
II	22 (57.9%)	20 (55.6%)		15 (57.7%)	15 (57.7%)	
III	4 (10.5%)	5 (13.9%)		4 (15.4%)	3 (11.5%)	
IV	2 (5.3%)	0		1 (3.8%)	0	
ECOG PS (%)			0.151			1.000
0	24 (63.2)	20 (55.6)		16 (61.5)	16 (61.5)	
1	14 (36.8)	12 (33.3)		10 (38.5)	10 (38.5)	
2	0	4 (11.1)		0	0	
CEA level	85.1 (0.42–2239.0)	373.9 (0.66–12118.0)	0.213	35.5 (0.42–288.2)	50.4 (0.66–934.2)	0.716
CA 19-9 level	561.1 (1.28–4929.0)	348.6 (0.1–5893.0)	0.116	211.1 (1.39–2074.7)	247.1 (0.4–3510.1)	0.838
Ascites (%)			0.522			0.932
None	18 (47.4)	17 (47.2)		12 (46.2)	13 (50.0)	
Mild	15 (39.8)	10 (27.8)		11 (42.3)	9 (34.6)	
Moderate	3 (7.9)	4 (11.1)		1 (3.8)	1 (3.8)	
Massive	2 (5.2)	5 (13.9)		2 (7.7)	3 (11.5)	
Histology (%)			0.021			1.000
Well to moderately differentiated	2 (5.3)	10 (27.8)		2 (7.7)	2 (7.7)	
Poorly differentiated or SRC	36 (94.7)	26 (72.2)		24 (92.3)	24 (92.3)	

MIS-IP, the patients who underwent minimally invasive surgery followed by intraperitoneal and systemic chemotherapy; SC-only, the patients who underwent systemic chemotherapy only; ECOG PS, Eastern Cooperative Oncology Group performance status; SRC, signet ring cell carcinoma.

**Table 2:** Clinicopathologic data of the patients who underwent minimally invasive surgery conjoined with intraperitoneal plus systemic chemotherapy for gastric cancer with peritoneal carcinomatosis.

Serial Number	Age	Sex	PCI Score	Procedure at the Diagnosis of PC	Time to Conversion Surgery (month)	Type of Conversion Surgery	Morbidity Associated Gastrectomy	Number of IP Chemotherapy Cycles	PFS (Month)	OS (Month)
1	72	Male	18	LGJ				5	10	10
2	52	Male	22	DL				6	6	7
3	57	Female	3	LGJ				7	6	7
4	71	Male	14	LGJ				10	9	9
5	43	Female	3	LDG			None	17	19	19
6	75	Male	21	DL				3	3	13
7	81	Male	3	LDG			DS leakage		11	13
8	35	Female	10	LDG			None	26	14	19
9	43	Male	17	DL	24	LTG	None	72	39	51
10	43	Male	11	LTG, segmental resection of transverse colon			None	25	13	15
11	70	Male	15	DL	5	LDG	None	23	30	35
12	37	Male	21	DL				8	6	8
13	68	Female	5	LTG			None	20	13	19
14	41	Male	12	LGJ	6	LTG	EJ leakage	5	6	7
15	48	Female	23	DL, FJ				6	4	4
16	54	Male	17	LTG			Bleeding	39	18	34
17	63	Male	15	DL	4	LTG	Fluid collection	17	10	10
18	45	Female	2	RTG, Splenectomy, Adrenalectomy			None	25	26	26
19	60	Male	5	LDG			Afferent loop syndrome	8	11	13
20	42	Male	8	LDG			None	21	20	20
21	82	Male	14	LGJ				17	4	5
22	72	Male	6	LTG, Splenectomy			CVA	18	16	16
23	73	Female	2	LDG			None	8	12	12
24	61	Male	12	LGJ	6	LTG	None	14	11	11
25	65	Male	7	LTG				16	11	11
26	54	Female	20	DL				17	9	9

PCI, peritoneal cancer index; PC, peritoneal carcinomatosis; IP, intraperitoneal; PFS, progression-free survival; OS, overall survival; DL, diagnostic laparoscopy; LGJ, laparoscopic gastrojejunostomy; LDG, laparoscopic distal gastrectomy; DS leakage, duodenal stump leakage; LTG, laparoscopic total gastrectomy; RTG, robotic total gastrectomy; EJ leakage, esophagojejunostomy leakage; FJ, feeding jejunostomy; CVA, cardiovascular accident.

**Table 3:** Comparison of clinical outcomes between the two groups.

Variables	MIS-IP (*n* = 26)	SC Only (*n* = 26)	*p*
Procedure at diagnostic laparoscopy (%)			
Non-curative gastrectomy	12 (46.2)	2 (7.7)	0.002
Bypass surgery	6 (23.1)	2 (7.7)	0.124
Exploration only	7 (26.9)	1 (3.8)	0.021
Conversion surgery (%)	4 (15.4)	0	0.037
Surgery-related complications (%)			
IP port infection	1 (3.8)	0	0.313
Bleeding	1 (3.8)	0	0.313
Anastomosis/duodenal stump leakage	2 (7.7)	0	0.149
Ileus	2 (7.7)	0	0.149
Splenic infarction	2 (7.7)	1 (3.8)	0.552
CVA	1 (3.8)	0	0.313
Severe morbidity (CDC ≥ 3)	3 (11.5)	0	0.074
1st line SC agent (%)			
FOLFOX	19 (73.1)	18 (69.2)	0.760
XELOX	7 (26.9)	1 (3.8)	0.021
FP	0	7 (27.0)	0.004
Chemotherapy induced toxicity ^†^			
Hematopoietic	7 (26.9)	9 (34.6)	0.548
Hepatic / renal	2 (7.7)	2 (7.7)	1.000
Neurologic	9 (34.6)	8 (30.8)	0.768
Intestinal	13 (50.0)	9 (34.6)	0.262
Fatigue	14 (53.8)	13 (50.0)	0.781
Severe toxicity (Grade ≥ 3)	4 (15.4)	2 (7.7)	0.385

MIS-IP, the patients who underwent minimally invasive surgery followed by intraperitoneal and systemic chemotherapy; SC-only, the patients who underwent systemic chemotherapy only; CVA, cardiovascular accident; CDC, Clavien–Dindo classification; SC, systemic chemotherapy; FP, fluoropyrimidine-based drug plus cisplatin. ^†^ Chemotherapy induced toxicity was evaluated according to the common terminology criteria for adverse events version 3.0.

**Table 4:** Multivariate analysis of prognostic factors for progression-free survival in gastric cancer patients with peritoneal carcinomatosis.

Variable	Univariate Analysis		Multivariate Analysis	
	HR (95% CI)	*p*	HR (95% CI)	*p*
Age at diagnosis	1.000 (0.973–1.029)	0.981		
Sex	1.298 (0.628–2.638)	0.481		
IP chemotherapy	0.431 (0.218–0.852)	0.015	0.213 (0.095–0.477)	<0.001
BMI	1.007 (0.907–1118)	0.891		
ASA classification		0.058		
I	1.000 (reference)			
II	2.280 (0.972–5.348)			
III	0.798 (0.211–3.017)			
IV	2.640 (0.318–21.897)			
ECOG PS (≥2 versus ≤1)	1.937 (0.975–3.848)	0.042	3.689 (1.606–8.476)	0.002
CEA	0.999 (0.995–1.003)	0.619		
CA 19-9	1.000 (1.000–1.001)	0.438		
Ascites		0.016		0.011
None	1.000 (reference)		1.000 (reference)	
Mild	2.872 (1.213–6.802)		4.079 (1.614–10.306)	
Moderate	5.101 (1.059–24.574)		2.098 (0.373–11.800)	
Massive	1.496 (0.489–4.577)		4.396 (1.282–15.067)	
Histology		0.945		
WD or MD	1.000 (reference)			
PD or SRC	1.052 (0.249–4.449)			

HR, hazard ratio; CI, confidence interval; IP, intraperitoneal; ECOG PS, Eastern Cooperative Oncology Group performance status; WD, well differentiated; MD, moderately differentiated; PD, poorly differentiated; SRC, signet ring cell.

## Data Availability

The data presented in this study are available on request from the corresponding author.
